# Efficacy of a brief psychological intervention for adolescents with recent suicide attempt: A randomized clinical trial

**DOI:** 10.1192/j.eurpsy.2025.10065

**Published:** 2025-07-10

**Authors:** Ainoa García-Fernández, Manuel Couce-Sánchez, Jorge Andreo-Jover, Wala Ayad-Ahmed, María Teresa Bobes Bascarán, Maria Angeles Boti, Manuel Canal Rivero, Ana Isabel Cebriá, Benedicto Crespo-Facorro, Marina Díaz-Marsá, Verónica Fernández-Rodrigues, Sandra Gómez-Vallejo, Ana González-Pinto, Iria Grande, Noelia Iglesias Gutiérrez, Luis Jiménez-Treviño, Purificación López-Pena, Diego J. Palao, Ángela Palao-Tarrero, Anna Pedrola-Pons, Miguel Ruiz-Veguilla, Elizabeth Suarez-Soto, Alejandro de la Torre-Luque, Iñaki Zorrilla, Víctor Pérez, SURVIVE Group, Pilar A. Sáiz

**Affiliations:** 1 Department of Psychiatry, Universidad de Oviedo, Oviedo, Spain; 2 Instituto de Investigación Sanitaria del Principado de Asturias (ISPA), Oviedo, Spain; 3 Instituto Universitario de Neurociencias del Principado de Asturias (INEUROPA), Oviedo, Spain; 4 Servicio de Salud del Principado de Asturias (SESPA) Oviedo, Spain; 5 Centro de Investigación Biomédica en Red de Salud Mental (CIBERSAM), Instituto de Salud Carlos III, Madrid, Spain; 6 Hospital La Paz Institute for Health Research (IdiPAZ), Madrid, Spain; 7 Department of Legal Medicine, Psychiatry and Pathology, School of Medicine, Universidad Complutense de Madrid, Madrid, Spain; 8 Hospital Clínico San Carlos, Madrid, Spain; 9 Departament de Medicina, Facultat de Medicina i Ciències de la Salut, Universitat de Barcelona (UB), Barcelona, Spain; 10 Bipolar and Depressive Disorders Unit, Hospital Clinic de Barcelona, Barcelona, Spain; 11 Institut d’Investigacions Biomèdiques August Pi i Sunyer (IDIBAPS), Barcelona, Spain; 12 Institute of Neurosciences (UBNeuro), Barcelona, Spain; 13 Hospital Virgen del Rocío, Instituto de Biomedicina de Sevilla (IBIS), Sevilla, Spain; 14 Department of Psychiatry, Universidad de Sevilla, Sevilla, Spain; 15 Mental Health Service, Hospital Universitari Parc Taulí, Unitat Mixta de Neurociència Traslacional I3PT-INc-UAB, Sabadell, Barcelona, Spain; 16 Department of Clinical and Health Psychology, Faculty of Psychology, Universitat Autònoma de Barcelona, Cerdanyola del Vallès, Spain; 17 Department of Child and Adolescent Psychiatry and Psychology, Hospital Clinic of Barcelona, Institut d’Investigacions Biomèdiques August Pi i Sunyer (IDIBAPS), Barcelona, Spain; 18 Department of Psychiatry, Araba University Hospital, Universidad de País Vasco UPV/EHU, Vitoria-Gasteiz, Spain; 19 Bioaraba Health Research Institute, BIOARABA, Vitoria-Gasteiz, Spain; 20 Department of Psychiatry, Clinical Psychology and Mental Health, La Paz University Hospital, Madrid, Spain; 21 Department of Psychiatry and Forensic Medicine, Faculty of Medicine, Universitat Autònoma de Barcelona, Cerdanyola del Vallès, Spain; 22 Department of Psychiatry, Universidad Autónoma de Madrid (UAM), Madrid, Spain; 23 Faculty of Social Sciences, International University of Valencia, Valencia, Spain; 24 Mental Health Institute, Hospital del Mar, Barcelona, Spain; 25 Hospital del Mar Research Institute (IMIM), Hospital del Mar, Barcelona, Spain; 26 Universitat Pomepeu Fabra, Barcelona, Spain; 27 Institute of Neuropsychiatry and Addictions, Hospital del Mar, Barcelona, Spain

**Keywords:** adolescent, psychological intervention, randomized controlled trial, suicide attempt, treatment

## Abstract

**Background:**

Adolescents are at a heightened risk of suicide reattempts following hospital discharge, but few evidence-based interventions exist. This study evaluated the efficacy of the self-awareness of mental health (SAM) program combined with treatment as usual (TAU) versus TAU alone in reducing reattempts among high-risk adolescents.

**Methods:**

A randomized clinical trial was conducted across nine Spanish hospitals (January 2021–March 2024) with 261 adolescents (12–17 years) who had attempted suicide within the last 15 days. Participants were assigned to SAM + TAU (n=128) or TAU (n=133), with 12-month follow-up. The primary outcome was suicide reattempts within 12 months; secondary analyses examined time to reattempt and associated risk factors.

**Results:**

After 12-months, no significant differences were found in reattempt rates [22.6% (SAM) versus 27.8% (TAU); OR=0.610, 95%CI (0.321–1.151), p=0.127] or time to reattempt [HR=0.606, 95%CI (0.390–1.021), p=0.060]. In SAM, attentional impulsivity emerged as a significant risk factor [HR=1.126, 95% CI (1.004–1.263), p=0.043], while nonplanning impulsivity was protective [HR=0.878, 95%CI (0.814–0.948), p<0.001]. In TAU, increased suicide risk was linked to suicidal intentionality [HR=1.341, 95%CI (1.009–1.782), p=0.044] and more prior attempts [HR=1.230, 95%CI (1.039–1.457), p=0.016]. Conversely, fewer psychiatric diagnoses emerged as a protective factor [HR=0.821, 95%CI (0.677–0.996), p=0.045].

**Conclusions:**

While no significant differences were found between groups, SAM identified important psychological factors influencing suicide risk. These findings provide a foundation for targeted interventions to prevent reattempts in adolescents.

## Introduction

Suicide is a growing risk among adolescents worldwide, positioning itself as a leading cause of death in this population [1, 2]. In parallel, there has been an increase in emergency department visits for suicide attempts among adolescents, particularly females, in recent years [3, 4]. This trend has been especially pronounced since the COVID-19 pandemic, which has led to a doubling of pediatric emergency department visits for mental health issues, including a five-fold increase in visits related to suicide [5, 6]. The period immediately following a suicide attempt, whether after an emergency department visit or psychiatric discharge, is a critical phase of heightened vulnerability and presents elevated risk for subsequent suicide [7, 8]. Psychological interventions that address the urgent needs of adolescents during this stage are essential for preventing future attempts.

Despite advances in the treatment of suicidal behaviors, there are still limited psychological interventions with sufficient empirical support for critical periods of high suicide risk [9]. Traditional psychological interventions, such as dialectical behavior therapy (DBT), are effective in reducing suicide attempts in adolescents [10–13]. However, cost and a shortage of trained therapists hinder timely and accurate implementation [14]. Furthermore, prompt psychological interventions in the critical weeks following hospital discharge may play a crucial role in determining long-term reductions in suicide risk [9]. In addition, being treated in the emergency room during the pandemic was associated with diminished risk of suicide, confirming the importance of brief interventions [15]. Consequently, independent research groups have developed brief, targeted interventions specifically designed for the high-risk transition period following hospital discharge and during emergency department visits [16–23]. However, these interventions have not demonstrated a significant advantage over standard care. While the number of suicide attempts decreased across all adolescents from baseline to the end of treatment, those groups reported no significant differences between the intervention and control groups. Few researchers have conducted randomized trials among adolescents at high risk of suicide, and, to our knowledge, none in Spain. Moreover, no brief, easily implemented, evidence-based psychological interventions have demonstrated clear efficacy for adolescents who have attempted suicide.

To address this urgent need in clinical practice, we developed a brief outpatient intervention called self-awareness of mental health (SAM) to reduce suicide attempts in adolescents after hospital discharge. The SAM intervention aims to enhance mental health awareness by focusing on both risk and protective factors associated with suicide [24]. We present the results of the first multicenter, randomized controlled trial of a clinic-based intervention and suicidal behaviors (SAM) in adolescents in Spain. The main objective of this study was to evaluate the efficacy of this brief intervention program for suicidal adolescents in reducing suicide reattempts, compared to the standard treatment provided in outpatient mental health clinics. The second aim was to determine the intervention’s impact on the time to suicide attempt recurrence over the 12-month follow-up. In addition, this study aimed to identify specific psychological and clinical profiles that may benefit more from the SAM intervention and the role of those profiles in suicide risk. A priori, we hypothesized that the SAM intervention would be associated with fewer suicide attempts and a longer time to suicide attempt recurrence over the 12-month follow-up period. Furthermore, the study explored whether specific psychological and clinical profiles might be associated with greater benefit from the SAM intervention, leading to a more significant reduction in suicide risk.

## Methods

### Trial design

This longitudinal study (trial registration: NCT04343703), conducted from January 2021 to March 2024, is part of the SURVIVE project (The Suicide Prevention and Intervention Study: Study Protocol for a Multisite Cohort Study with Nested Randomized-Controlled Trials) [24]. It focuses specifically on adolescents, representing a subsample of the general SURVIVE cohort. SURVIVE is the largest nationwide project in Spain, conducted by nine research centers: Hospital Clinic (Barcelona), Corporació Sanitaria Parc Taulí (Barcelona), Hospital Universitario La Paz (Madrid), Hospital Clínico (Madrid), Hospital Santiago de Áraba (Vitoria), Hospital Río Hortega (Valladolid), Hospital de Valdecilla (Cantabria), Hospital Virgen del Rocío (Sevilla), and Hospital Central Universitario (Asturias).

The sample size was determined a priori based on a two-arm parallel-group randomized controlled trial with a 12-month follow-up, analyzed using repeated-measures Analysis of Covariance (ANCOVA) adjusted for baseline scores [25]. Calculations assumed a two-sided alpha level of 0.05, statistical power of 80% (β = 0.20), an expected standardized effect size (Cohen’s d) of 0.375, and a between-wave correlation (ρ) of 0.30. Under these assumptions, the estimated sample size required to detect a statistically significant between-group difference was 94–95 participants per group. To account for an anticipated 30% attrition rate, we targeted a final sample size of 135 participants per group, yielding a total sample of 270 participants.

This multicenter study is a single-blind randomized controlled trial. Following completion of the baseline assessment, participants were assigned to one of the two treatment groups according to a 1:1 algorithm. We used ePRO, an online randomization service, to generate the assignment. Allocation concealment was guaranteed, as the randomization code was only released once a participant had been fully enrolled, which occurred only after baseline data collection. The two psychological intervention conditions were the SAM program + treatment as usual (TAU) or TAU alone. Due to the nature of the psychological interventions, blinding of participants and clinical staff was not feasible [24]. Patients or members of the public were not involved in the design, conduct, or reporting of this study.

The present study was conducted in accordance with international guidelines for reporting randomized controlled trials: the CONSORT 2025 guidelines [26] and the NIH Quality Assessment Tools for Controlled Intervention Studies [27] (Supplementary Tables S1 and S2).

### Participants

We recruited patients who presented to the emergency department using the following inclusion criteria: adolescents aged 12–17 years who had attempted suicide within the past 15 days and received treatment in the emergency department of their respective hospitals. Both adolescents and their parents were willing and able to comply with the study procedures and to provide written informed consent. Exclusion criteria included incapacity to give informed consent, lack of fluency in Spanish, and current participation in another clinical study likely to interfere with the objectives of the SURVIVE study.

The ethics committee for human research at each recruiting site approved all SURVIVE study protocols. The study adheres to both national and international ethical guidelines, including the most recent version of the World Medical Association’s Declaration of Helsinki (2013). In compliance with Spanish and European Union regulations, the project received approval from the Juvenile Prosecution Service. All participants, along with their parents or legal guardians, provided written informed consent prior to enrollment.

### Data collection and assessments

Trained psychologists and psychiatrists assessed participants in the 15 days following their admission to the emergency department due to a suicide attempt. The timing and setting of the baseline assessment varied depending on whether the participant was hospitalized. For nonhospitalized individuals, the baseline assessment was conducted at their mental health center within 15 days of the attempt. Hospitalized participants were assessed during hospitalization (if psychiatrically stable) or shortly postdischarge, based on their condition and preferences. Assessments were conducted at two points in time: baseline and 12 months, and the same trained professionals conducted both assessments. The baseline (V0) interview was face to face and, when possible, so was the 12-month interview. If an in-person interview was not feasible, we conducted it by telephone, supplemented by a review of electronic clinical records to verify information, including details of the suicide attempt, such as the dates and methods. Importantly, the clinical psychologists who conducted the SAM intervention were different from those providing TAU, and the clinicians responsible for outcome assessment were aware of the participants’ group allocation. It is important to note that the main outcome, suicide attempt (yes/no) and its timing, is objectively and reliably measurable, independently of whether the assessors were aware of the participants’ group allocation. Given the nature of the interventions, blinding of participants and staff to treatment allocation was not possible.

The baseline assessment consisted of an ad hoc questionnaire to gather sociodemographic information, including age, sex, religion, migration status, current academic year, course repetition, and parental education. In addition, we collected clinical data using the Spanish versions of the following psychometric scales:

- The Mini International Neuropsychiatric Interview for Children and Adolescents (MINI-KID), version 7.0.2, [28] is a structured clinical interview designed to assess psychiatric disorders in children and adolescents. It includes a series of standardized questions based on criteria of the Diagnostic and Statistical Manual of Mental Disorders, Fourth Edition, and the International Statistical Classification of Diseases and Related Health Problems, 10th Revision. Psychiatric diagnoses were grouped into the following categories: (1) affective disorders (including major depressive episode, dysthymic disorder, and mood dysregulation disorder); (2) anxiety disorders (including panic disorder, agoraphobia, social anxiety disorder, obsessive-compulsive disorder, posttraumatic stress disorder, and generalized anxiety disorder); (3) alcohol-related disorders (alcohol abuse and dependence); (4) substance-related disorders (drug abuse and dependence); (5) attention-deficit/hyperactivity disorder; (6) disruptive behavior disorders (conduct disorder and oppositional defiant disorder); (7) psychotic disorders; (8) eating disorders (anorexia nervosa and bulimia nervosa); and (9) adjustment disorders.
- The Columbia Suicide Severity Rating Scale [29, 30] is a clinician-administered rating scale to assess the severity and risk of suicidal thoughts and behaviors. Suicidal ideation is rated on a Likert-type scale from 1 to 5, with scores from 1 to 3 indicating the absence of intentionality and scores from 4 to 5 indicating the presence of intentionality. Suicidal behaviors included both the total number of suicide attempts and nonsuicidal self-injurious behaviors, assessed in a binary way (yes/no). The lethality of suicide attempts is assessed on a 6-point ordinal Likert scale from 0 (minor physical damage) to 5 (death). Additionally, attempts are categorized as <2 (minor physical damage or low suicide attempt lethality) or ≥ 2 (moderate physical damage to lethal outcome requiring medical attention or high suicide attempt lethality), following criteria established in recent studies [31, 32].
- The Patient Health Questionnaire-9 (PHQ-9) [33, 34] is a self-report screening instrument to assess the severity of depressive symptoms over the past two weeks. It includes nine items that assess the frequency of depressive symptoms within this timeframe. Each item is rated on a 4-point Likert scale ranging from 0 (not at all) to 3 (almost every day). The total severity score ranges from 0 to 27, with higher scores indicating greater clinical severity of depressive symptoms.
- The Strengths and Difficulties Questionnaire [35, 36] is a self-report scale used to evaluate behavioral and emotional difficulties, as well as assess strengths in children and adolescents. It consists of 25 items divided evenly into five subscales: emotional problems, conduct problems, hyperactivity/inattention, peer relationship problems, and prosocial behavior. Each item is rated on a 3-point Likert scale ranging from 0 (not true) to 2 (certainly true). The total difficulties score, calculated by summing the emotions, conduct, inattention-hyperactivity, and peer problems scales, ranges from 0 to 40, with higher scores indicating more problems.
- The Barratt Impulsiveness Scale (BIS-11) [37, 38] is a self-report scale used to assess trait impulsivity. It consists of 30 items grouped into three subscales: attentional, motor, and non-planning impulsivity. Each item is scored on a 4-point Likert scale ranging from 1 (rarely/never) to 4 (almost always). A total score is also calculated.
- The EuroQoL 5 Dimensions – 5 Levels [39, 40] is a health-related quality-of-life measure consisting of five dimensions: mobility, self-care, daily activities, pain/discomfort, and anxiety/depression. Each dimension has five response levels, ranging from 1 (no problems) to 5 (extreme problems). The level sum score (LSS) is a total score ranging from 5 (no problems) to 25 (extreme problems), indicating overall health status with higher scores reflecting worse quality of life [41]. Additionally, a visual analogue scale (VAS) is used to assess overall health on a scale from 0 (worst imaginable health) to 100 (best imaginable health).

### Psychological interventions

Treatment as usual (TAU) consists of the routine treatment procedures implemented at each participating site. While TAU may vary across sites, it typically includes a combination of case management strategies – such as telephone follow-ups and visits by mental health services – and pharmacotherapy. In this study, any nonspecific intervention targeting suicidal behavior or suicide prevention is classified as TAU. All participants received TAU as part of their care.

The SAM intervention is a postdischarge outpatient program consisting of a brief individual psychological intervention specifically designed for adolescents who have recently attempted suicide. The sessions were conducted by psychiatrists and clinical psychologists with specific training in this intervention. SAM is based on the Youth Aware of Mental Health (YAM), a manualized, universal primary prevention tool for suicide, originally developed for the Saving and Empowering Young Lives in Europe (SEYLE) study [42]. SAM employs a third-generation individual psychological approach aimed at increasing mental health awareness about risk and protective factors associated with suicide. Unlike other interventions such as DBT, which require extensive training and prolonged treatment duration, SAM is designed as a brief, adaptable program that can be implemented in outpatient settings with a relatively low-resource burden. Its structure is inspired by psychoeducational components of programs such as YAM but is adapted to the specific clinical needs of adolescents with recent suicide attempts. It aims at boosting mental health literacy (i.e., knowledge about depression and anxiety) and enhances skills for coping with adverse life events, stress, and suicidal behaviors, including the development of a safety plan. The ultimate goal of this intervention is to prevent the recurrence of suicide attempts.

The intervention consists of five individual weekly sessions, each lasting approximately 60 minutes, conducted in a clinical setting. The program is designed specifically for adolescents, emphasizing their active engagement in shaping a personal life project. It fosters a collaborative environment where the adolescent takes a central, proactive role. All sessions are conducted in person, ensuring direct interaction and active participation. The structure of the sessions is in Tables 1 and 2.

**Table 1.** General structure of the SAM

	Goals	Procedures
Session I. Psychoducational Intervention	Problem assessment and psychoeducation	Problem assessment is conducted through a structured initial clinical interview guided by the psychoeducational manual “Know How You Feel and Try to Improve It.” It includes six chapters: Mental health awareness Self-help tips Stress and crisis Depression and suicidal thoughts Warning signs Safety plan: who to contact Introduction to the development of a safety plan, which will be addressed throughout subsequent sessions
Session II. Role playing I	Awareness about decisions	Focus on understanding and motivating adolescents to comprehend their own decisions.
Session III. Role playing II	Awareness about feelings and handling stress and crisis situations	Address aspects related to stress and identify potential sources of stress
Session IV. Role playing III	Awareness about depression and suicidal thoughts and behaviors	Discuss the concept of depression, its characteristic symptoms, and suicidal thoughts
Session V. Closing session	Warning signs and getting help – who to contact?	Summarize topics discussed in previous sessions, review expressed problems, explore how situations can affect individuals, discuss alternative problem-solving methods, and reflect on how the adolescent’s approach to dealing with adversities has evolved throughout the program The closing session aims to create a positive and encouraging atmosphere, fostering optimism about the future for the adolescent

*Note*: Manuals describing the intervention – including the therapist manual and the patient manual – are available upon request.

**Table 2.** General structure of the five sessions that make up the intervention

	**Session 1**Introduction mental health awareness	**Session 2**Awareness about decisions	**Session 3**Awareness of feelings	**Session 4**Awareness about depression and suicidal thoughts	**Session 5**Warning signs. Closure
1ª part 10–15 min	Presentation and introduction to the adolescent manual	Review and revision of homework	Review and revision of homework	Review and revision of homework	Working with the metaphor of the rider
2ª part 20–25 min	Exploring problems. Garden metaphor	Theoretical content and role playing I	Theoretical content and role playing II	Theoretical content and role playing III	Warning signs and use of the safety plan
3ª part 5–10 min	Security plan	Ability 1 Safe place	Ability 2 Relaxation techniques	Ability 3 problem solving	Recapitulation
4ª part 5–10 min	Closure and assignment of task	Closure and assignment of task	Closure and assignment of task	Closure and assignment of task	Final closure and assignment of follow-ups

*Note*: Manuals describing the intervention – including the therapist manual and the patient manual – are available upon request.

Manuals describing the intervention – including the therapist manual and the patient manual – are available upon request.

### Outcome measures

The primary outcome was the occurrence of a subsequent suicide attempt within a 12-month follow-up period. In addition to participant assessments, medical records were reviewed to complement and confirm the information obtained.

Secondary outcomes included sociodemographic and clinical characteristics, as well as variables such as suicidal ideation and behavior, depression, impulsivity, emotional and behavioral difficulties, personal strengths, and quality of life. These factors were examined to explore their potential associations with the recurrence of suicide attempts and the time to recurrence over the 12-month follow-up period.

### Statistical analyses

We report baseline descriptive statistics of frequencies and percentages for categorical variables and means and standard deviations (SDs) for continuous variables. Prior to conducting bivariate analyses, we assessed the normality of continuous variables using the Kolmogorov–Smirnov test. When variables showed significant deviations from normality (p < 0.05), nonparametric tests (Mann–Whitney U test) were used for comparisons between groups of continuous variables. Chi-square tests were used for comparisons involving categorical variables.

We estimated the cumulative distribution of time to a new suicide attempt using the Kaplan–Meier method. Differences in time to reattempt between groups were evaluated using a log-rank test. We estimated hazard ratios (HRs) and 95% confidence intervals (CIs) based on a Cox proportional hazards regression model, with treatment as a predictor. To address possible attrition bias, missing follow-up data were cross-validated using electronic health records when available.

We set a two-tailed significance level of 0.05.

All statistical analyses were conducted using SPSS version 27.0 and JASP version 0.19.1.

## Results

### Baseline characteristics

A total of 270 patients (235 females, 87.0%) completed the baseline assessment. Of these, 261 adolescents (mean = 15.00, SD = 1.52; 227 females [87.0%]) completed the 12-month follow-up. Specifically, 133 participants (97.79%) were randomly assigned to the TAU group (87.0% female; mean = 14.97, SD = 1.56) and 128 participants (95.52%) from the SAM group (86.70% female; mean = 15.04, SD = 1.49) completed the follow-up assessment (see Table 3).

**Table 3.** Sociodemographic features of the sample

	Total sample, N = 261	TAU group, n = 133	SAM group, n = 128	Statistics (p)
Age [mean (SD)]	15.00 (1.52)	14.97 (1.56)	15.04 (1.49)	U = 8340.000, p = 0.774
Sex, n (%) Female Male	227 (87.0) 34 (13.0)	116 (87.2) 17 (12.8)	111 (86.7) 17 (13.3)	χ^2^ = 0.014, p = 0.905
Religion, n (%) Yes No Other	92 (35.5) 161 (62.2) 6 (2.3)	47 (35.3) 84 (63.2) 2 (1.5)	45 (35.7) 77 (61.1) 4 (3.2)	χ^2^ = 0.826, p = 0.662
Migration, n (%) No Yes	210 (80.5) 51 (19.5)	108 (81.2) 25 (18.8)	102 (79.7) 26 (20.3)	χ^2^ = 0.095, p = 0.758
Current academic year, n (%) Primary 1° Secondary 2° Secondary 3° Secondary 4° Secondary 1° Bachelor 2° Bachelor V ocational training Drop school	3 (1.1) 23 (8.8) 44 (16.9) 56 (21.5) 65 (24.9) 27 (10.3) 25 (9.6) 15 (5.7) 3 (1.1)	0 (0) 14 (10.5) 22 (16.5) 29 (21.8) 31 (23.3) 14 (10.5) 14 (10.5) 9 (3.4) 0 (0.0)	3 (2.3) 9 (7.0) 22 (17.2) 27 (21.1) 34 (26.6) 13 (10.2) 11 (8.6) 6 (2.3) 3 (2.3)	χ^2^ = 8.201, p = 0.414
Repeated courses, n (%) No Yes	193 (73.9%) 68 (26.1%)	108 (81.2%) 25 (18.4%)	85 (66.4%) 43 (33.6%)	χ^2^ = 7.413, p = **0.006**
Parental education level, n (%) None Primary Secondary University Unknown	3 (1.1) 14 (5.4) 122 (46.7) 98 (37.5) 24 (9.2)	3 (2.3) 7 (5.3) 55 (41.4) 53 (39.8) 15 (11.3)	0 (0) 7 (5.5) 67 (52.3) 45 (35.2) 9 (7.0)	χ^2^ = 6.240, p = 0.182

SAM, self-awareness of mental health; SD, standard deviation; TAU, treatment as usual.

The values in bold are the significant values (p < 0.05).

**Figure 1.**
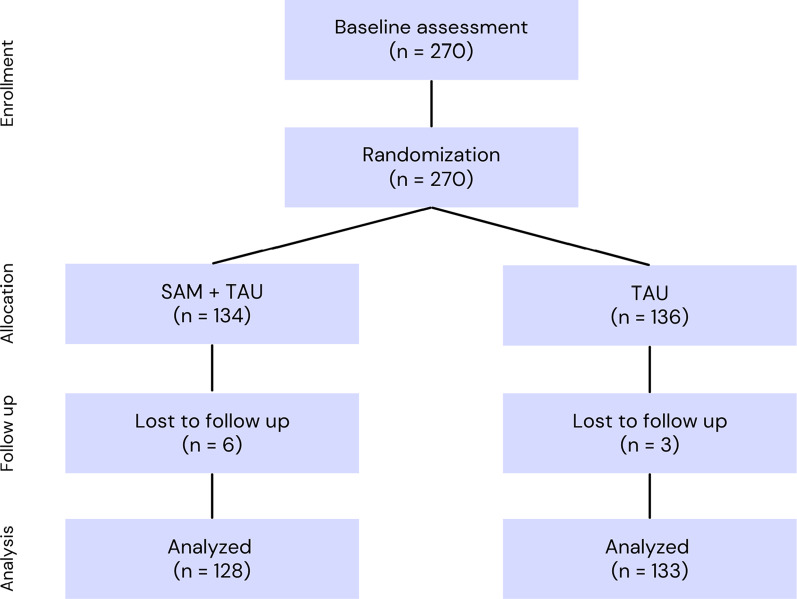
Flowchart. SAM, self-awareness of mental health; TAU, treatment as usual.

The number of withdrawals at follow-up was low and did not differ significantly between groups (TAU: 3 (2.20%) of 136; SAM: 6 [4.47%] of 134; χ^2^ = 1.08, p = 0.298) (see Figure 1). As the continuous variables showed significant deviations from normality based on the Kolmogorov–Smirnov test (p < 0.05), all between-group comparisons involving continuous data were conducted using the nonparametric Mann–Whitney U test. Both groups were predominantly females (approximately 87%), with a mean age of 15 years, and around 80% were indigenous. However, we found a significant difference in the proportion of adolescents who had repeated a school year (p = 0.006), with a higher prevalence in the SAM group. In terms of clinical characteristics, both groups had an average of three psychiatric diagnoses, and all adolescents had prescriptions for at least one pharmacological treatment, with antidepressants the most common (50%). Notably, adolescents in both groups exhibited mean PHQ-9 scores, indicative of moderately severe depression, and showed comparable levels across the three domains of impulsivity, as measured by the BIS-11. The only significant difference emerged in conduct problems (p = 0.032), with adolescents in the TAU group showing higher scores than those in the SAM group. Regarding suicidal behavior, we observed no significant differences between the two groups. Approximately three-quarters of adolescents in both groups reported engaging in nonsuicidal self-injurious behaviors. The predominant method of suicide attempt was drug overdose, and around 50% of adolescents made attempts of moderate to severe severity, necessitating medical attention (see Tables 3 and 4).

**Table 4.** Clinical and suicidal features of the sample

Mean (SD)	Total sample, *N* = 261	TAU group, n = 133	SAM group, n = 128	Statistics (p)
Number of Psychiatric Diagnoses (MINI-KID)	3.19 (2.18)	3.04 (1.99)	3.35 (2.36)	U = 8090.000, p **=** 0.481
Depression (PHQ–9)	17.85 (5.60)	17.92 (5.75)	17.77 (5.46)	U = 8382.500, p = 0.832
EQ–5D–5L (LSS)	10.31 (3.24)	10.42 (3.43)	10.20 (3.04)	U = 8395.500, p = 0.847
EQ–5D–5L (VAS)	47.77 (21.72)	47.10 (22.05)	48.48 (21.43)	U = 8208.000, p = 0.617
Impulsivity (BIS–11) Total Attentional Motor Nonplanning	60.48 (12.79) 19.53 (4.55) 19.13 (6.76) 21.81 (6.80)	61.48 (15.37) 19.35 (4.49) 19.39 (6.32) 22.44 (6.51)	59.43 (13.18) 19.40 (4.63) 18.87 (7.21) 21.16 (7.06)	U = 7565.500, p = 0.120 U = 8219.000, p = 0.630 U = 8052.000, p = 0.450 U = 7720.500, p = 0.194
SDQ Emotional problems Conduct problems Hyperactivity Peer problems Prosocial behavior Total	7.05 (1.89) 3.75 (2.16) 6.02 (2.04) 3.68 (2.25) 8.01 (1.91) 20.51 (5.25)	7.07 (1.84) 4.02 (2.15) 6.08 (2.10) 3.68 (2.23) 8.05 (1.81) 20.85 (5.26)	7.04 (1.95) 3.47 (2.14) 5.96 (1.99) 3.68 (2.29) 7.97 (2.02) 20.15 (5.23)	U = 8466.500, p = 0.940 U = 7220.000, **p = 0.032** U = 8091.000, p = 0.485 U = 8464.000, p = 0.937 U = 8511.500, p = 0.999 U = 7832.500, p = 0.264
Number of drugs	1.14 (1.09)	1.08 (1.09)	1.20 (1.08)	U = 7956.000, p = 0.338
Drug type, n (%yes) Antidepressant Benzodiazepines Antipsychotics Others	129 (49.4) 74 (28.4) 64 (24.5) 28 (10.7)	68 (51.1) 36 (27.1) 29 (21.8) 11 (8.3)	64 (50.0) 38 (29.7) 35 (27.3) 17 (13.3)	χ^2^ = 0.033, p = 0.855 χ^2^ = 0.220, p = 0.639 χ^2^ = 1.081, p = 0.298 χ^2^ = 1.710, p = 0.191
Number of previous attempts	2.31 (2.07)	2.31 (1.97)	2.31 (2.18)	U = 8501.500, p = 0.985
NSSI No Yes	65 (24.9%) 196 (75.1%)	33 (24.8%) 100 (75.2%)	32 (25.0%) 96 (75.0%)	χ^2^ = 0.001, p **=** 0.972
Ideation intentionality (0–5), n (%yes)	3.73 (1.46)	3.63 (1.51)	3.83 (1.41)	U = 7854.000, p **=** 0.257
Ideation Intentionality (1–3 vs. 4–5), n (%yes)	171 (68.7)	87 (69.0)	84 (68.3)	χ^2^ = 0.016, p = 0.898
Medical damage (%severe ≥2), n (%yes)	128 (52.7)	61 (50.4)	67 (54.9)	χ^2^ = 0.495, p = 0.482
Methods, n (%yes) Poisoning with solid or liquid Poisoning by other gases or vapors Cutting Instrument Savings, strangulation, or suffocation Jumping from a high building Others	209 (80.1) 4 (1.5) 29 (11.1) 4 (1.5) 9 (3.4) 6 (2.3)	106 (79.7) 4 (3.0) 16 (12.0) 2 (1.5) 3 (2.3) 2 (1.5)	103 (80.5) 0 (0) 13 (10.2) 2 (1.6) 6 (4.7) 4 (3.1)	χ^2^ = 5.926, p = 0.313
Family history of suicide, n (%yes)	61 (23.4)	31 (23.3)	30 (23.4)	χ^2^ = 0.001, p = 0.980

Abbreviations: BIS, Barratt Impulsiveness Scale; EQ-5D-5L, EuroQol 5 Dimensions – 5 Levels; LSS, level of severity scoring; MINI-KID, Mini International Neuropsychiatric Interview for Children and Adolescents; NSSI, nonsuicidal self-injury; PHQ-9, Patient Health Questionnaire-9; SAM, self-awareness of mental health; SD, standard deviation; SDQ, Strengths and Difficulties Questionnaire; TAU, treatment as usual; VAS, visual analogue scale.

### Efficacy results

After a 12-month follow-up period, the proportion of individuals with suicide reattempts did not differ significantly between the SAM group (29 [22.6%] and the TAU group 37 [27.8%]; odds ratio = 0.610, 95% CI [0.321–1.151], p = 0.127). We found no significant differences in the total number of new suicide attempts among those who reattempted (U = 518.5, p = 0.775), with 52 events in 29 patients in the intervention group (mean = 1.79) compared to 77 events in 37 patients in the control group (mean = 2.08).

**Figure 2.**
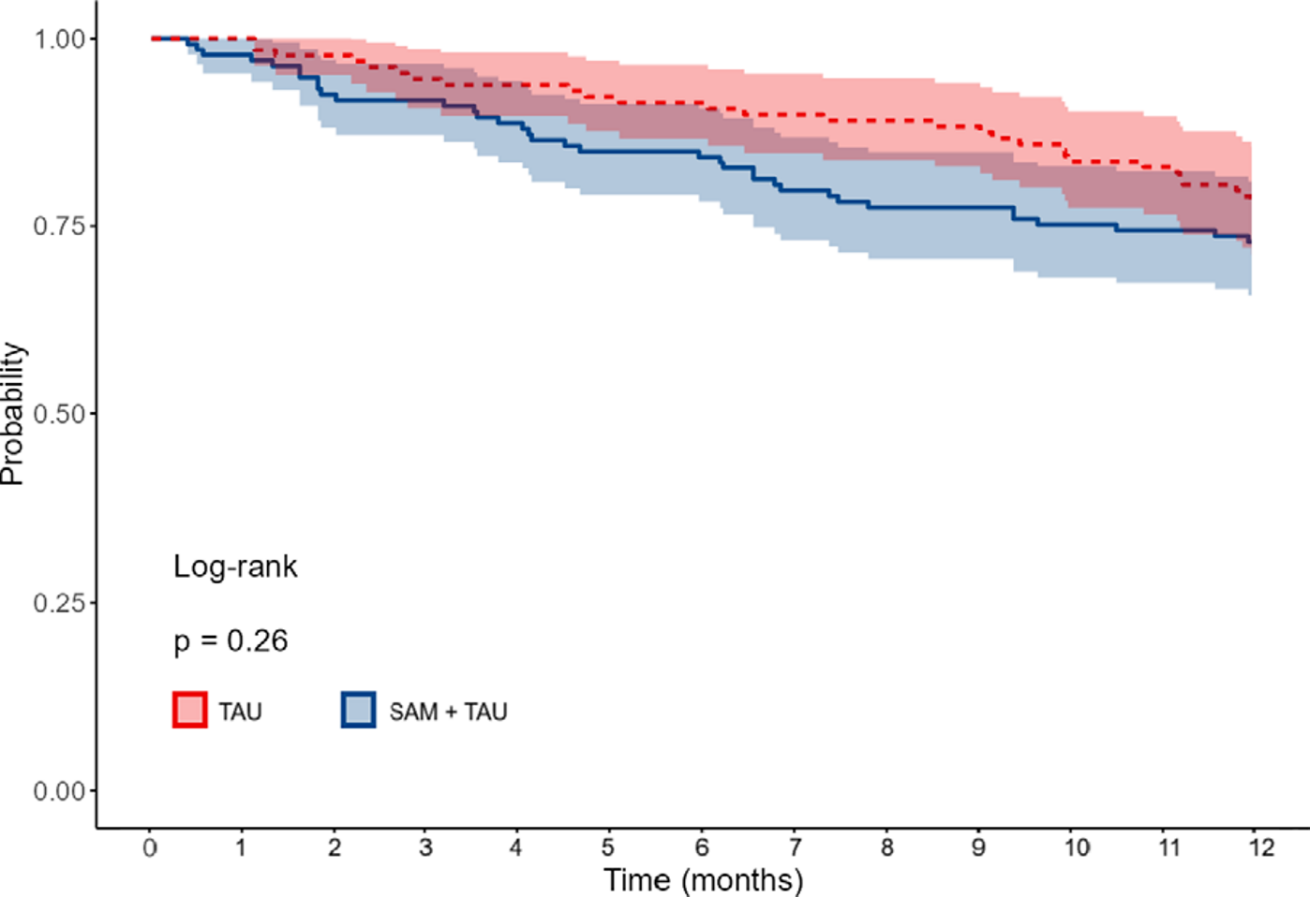
Survival curve. SAM, self-awareness of mental health; TAU, treatment as usual.

The Kaplan–Meier survival curve in Figure 2 illustrates the time to suicide attempt recurrence over the follow-up period. We used a Cox proportional hazards regression analysis to examine the intervention’s impact on the time to suicide attempt recurrence over the 12-month follow-up. The model included the same predictors as the logistic regression analysis. We found no significant differences in time to suicide attempt between the SAM and TAU groups (HR = 0.606, 95% CI [0.360–1.021], p = 0.060). The presence of suicidal intentionality (HR = 1.322, 95% CI [1.065–1.641], p = 0.011) and a higher number of prior attempts (HR = 1.134, 95% CI [1.016–1.267], p = 0.008), as well as attentional impulsivity (HR = 1.089, 95% CI [1.014–1.171], p = 0.014) were associated with a higher risk of recurrence during the follow-up period. Nonplanning impulsiveness was identified as a protective factor (HR = 0.932, 95% CI [0.889–0.976], p = 0.003). Other predictors were not significantly associated with time to suicide attempt recurrence (Table 5).

**Table 5.** Results of primary and outcome for the risk of repeat suicide attempt in the treatment groups and covariates

Predictor	Hazard ratio	95% CI	p-value
Clinical trial arm (SAM)	0.606	0.360–1.021	0.060
Migration (yes)	0.811	0.404–1.628	0.556
Gender (male)	0.769	0.319–1.859	0.560
Age	0.901	0.760–1.069	0.231
Repeated courses (yes)	1.295	0.665–2.520	0.447
PHQ–9	1.002	0.933–1.076	0.959
EQ–5D–5L (LSS)	0.996	0.982–1.011	0.618
EQ–5D–5L (VAS)	1.022	0.916–1.141	0.699
BIS–11 (attentional subscore)	**1.089**	**1.014–1.171**	**0.019**
BIS–11 (motor subscore)	1.028	0.981–1.076	0.244
BIS–11 (nonplanning subscore)	**0.932**	**0.889–0.976**	**0.003**
SDQ (emotional problem)	0.849	0.711–1.015	0.072
SDQ (conduct problem)	1.009	0.876–1.161	0.906
SDQ (hyperactivity)	0.954	0.815–1.117	0.560
SDQ (peer problem)	1.000	0.887–1.129	0.995
SDQ (prosocial)	0.886	0.778–1.007	0.065
Ideation intentionality	**1.322**	**1.065–1.641**	**0.011**
Number of previous attempts	**1.134**	**1.016–1.267**	**0.026**
Number of psychiatric diagnoses (MINI-KID)	0.905	0.794–1.031	0.135

Abbreviations: BIS-11, Barratt Impulsiveness Scale; EQ-5D-5L, EuroQol 5 Dimensions – 5 Levels; LSS, level of severity scoring; MINI-KID, Mini International Neuropsychiatric Interview for Children and Adolescents; PHQ-9, Patient Health Questionnaire-9; SDQ, Strengths and Difficulties Questionnaire; VAS, visual analogue scale.

The values in bold are the significant values (p < 0.05).

The presence of suicidal intentionality (HR = 1.341, 95% CI [1.009–1.782], p = 0.044) and a higher number of previous attempts (HR = 1.230, 95% CI [1.039–1.457], p = 0.016) stratified by groups within the TAU group, demonstrated significant associations, both emerging as risk factors for reattempts during follow-up. Additionally, a lower number of psychiatric comorbidities appeared to be a protective factor (HR = 0.821, 95% CI [0.677–0.996], p = 0.045). Conversely, in the SAM group, we found statistical significance only for attentional impulsivity as a risk factor (HR = 1.126, 95% CI [1.004–1.263], p = 0.043) and nonplanning impulsivity as a protective factor (HR = 0.878, 95% CI [0.814–0.948], p < 0.001).

## Discussion

This research represents the first multicenter randomized controlled trial of a psychological intervention for reducing suicide reattempt among adolescents at high suicide risk in Spain. Importantly, the baseline characteristics of the participants in both the SAM and TAU groups were comparable, ensuring that the observed outcomes are reflective of the intervention effects rather than preexisting differences. SAM was not significantly more effective in preventing suicide reattempts during a 12-month follow-up period. Several factors may explain this result, including the brief duration of the SAM intervention and the high adherence to TAU observed in both groups. It is possible that a more extended intervention or a combination of SAM with additional support strategies could yield stronger effects in reducing reattempts. On the other hand, the rate of suicide attempt and the proportion of participants reattempting suicide in the SAM group were slightly lower than in the TAU group. Interestingly, impulsivity appeared to influence the effectiveness of SAM. Nonplanning impulsivity emerged as a protective factor for patients in the SAM group, while attentional impulsivity was a risk factor. Notably, no other factors were associated with repeated suicide attempts in the SAM group. In contrast, in the TAU group, only the number of previous suicide attempts and the intentionality of suicidal ideation were significantly linked to reattempts. It is important to highlight that we conducted the study during the COVID-19 pandemic, a health emergency that saw a general increase in suicidal behaviors across the adolescent population [43]. Despite this challenging context, the study may offer some initial evidence that the SAM intervention has potential to reduce suicide attempts among adolescents at high suicide risk.

Findings from this study align with previous research on brief psychological therapies, where interventions demonstrated a promising clinical trend but did not yield statistically significant differences in outcomes. Specifically, our findings are consistent with those from the first brief intervention designed to reduce suicide attempts following hospital discharge [16]. SAM did not demonstrate a statistically significant effect on the frequency of suicide attempts or the time to an attempt. However, despite the lack of clinical effects, the results agree with the hypothesis. Over the 12-month follow-up, the SAM group showed larger improvements, offering preliminary evidence of clinical significance and extending the trends observed in Kennard’s study over shorter follow-up periods of 1, 3, and 6 months postdischarge. The lack of differential effects raises concerns. Similarly, a recent trial of an ultrabrief suicide crisis intervention based on Interpersonal Psychotherapy for Adolescents (IPT-A-SCI) also reported clinical improvements, including reductions in suicidal ideation and behavior, depression, and anxiety. However, no significant differences emerged between the intervention, TAU, and waitlist control groups [44]. Some studies have suggested that inconsistent training of the therapeutic team could be an explanation [45]. However, we dismiss this possibility because the team conducting the interventions, composed of both psychiatrists and clinical psychologists, underwent structured and uniform training. They also held monthly meetings to address challenges and resolve any issues, ensuring consistent practice and cohesion between the research centers.

Finally, we found significant predictors of reattempt during the 12-month follow-up period, specific to each group. As expected, in the TAU group, a history of previous suicide attempts emerged as a strong predictor of future attempts. The observed association between a higher number of prior attempts and increased odds of recurrence reinforces the well-established role of prior suicide attempts as a primary risk factor [8]. The risk is even greater for individuals with multiple prior attempts, as highlighted by a recent meta-analysis and systematic review across all age groups [46]. This phenomenon could be related to the development of an acquired capacity for suicide, driven by exposure and habituation to the physical and psychological pain associated with suicidal behavior [47]. Van Gerpen et al. [48] underscore that youth with a history of multiple suicide attempts are at a significantly increased risk of future attempts and require close monitoring, especially during the critical period immediately following discharge from a hospital or emergency department [49]. Moreover, the presence of suicidal intentionality also increased the probability of suicide reattempt. This finding aligns with previous research demonstrating that prior suicidal ideation significantly increases the risk of all suicide-related behaviors, including recurrent ideation, reattempts, and death [50]. Specifically, persistent suicidal thoughts following an attempt may further raise the probability of future attempts [46]. Furthermore, a lower number of psychiatric diagnoses were associated with a reduced risk of suicide reattempts. This finding complements existing evidence that psychiatric comorbidity is a significant predictor of suicidal behavior in adolescents [51]. Supporting this, a recent study by Szmajda et al. [52] highlights the clinical utility of including primary psychiatric diagnoses in suicide risk assessments among youth. In this context, the protective effect observed in adolescents with fewer diagnoses could reflect a less complex clinical profile and milder symptomatology, ultimately resulting in a lower likelihood of reattempt.

Regarding the SAM group, this study provides a nuanced perspective on the role of different dimensions of impulsivity in suicide risk. Although impulsivity was not a primary outcome and the study was not originally designed to explore its effects, we conducted exploratory analyses to investigate whether different impulsivity dimensions could be associated with suicide reattempts. These analyses, while limited, provide preliminary insights into potential cognitive and behavioral factors that may influence intervention outcomes. Specifically, attentional impulsivity emerged as a potential risk factor, suggesting that difficulties in sustaining attention and inhibiting reactive responses may increase vulnerability to suicide reattempts. This dimension of impulsivity could also interfere with the ability to fully benefit from psychoeducational interventions like SAM, which require sustained attention and cognitive engagement. Conversely, the protective role of nonplanning impulsivity indicates that a lack of long-term foresight might paradoxically act as a buffer against structured, deliberate suicidal actions. This aligns with the idea that suicide is rarely entirely impulsive [53] and underscores the multifaceted nature of impulsivity, with specific dimensions influencing suicide risk differently [54]. Importantly, a recent systematic review and meta-analysis [46] found that impulsivity, although it plays a significant role in initial suicide attempts, may have less influence on subsequent reattempts across all age groups. These findings suggest that tailoring interventions such as SAM for patients with specific cognitive and behavioral profiles, particularly impulsivity traits, may enhance their effectiveness. However, these observations should be interpreted with caution, given the exploratory nature of the analyses, and further research is needed to validate these findings and explore their clinical relevance.

### Limitations and strengths

This study has several limitations. First, data on patients assessed for eligibility were not available. Although all individuals meeting the inclusion criteria were invited to participate in the study in the emergency department, records exist only for those who agreed to participate. Those who declined were not documented. Second, the sample was predominantly female, limiting the generalizability. This gender imbalance is consistent with previous studies on adolescent samples [9]. The “sex paradox in suicide” explains this disparity: despite higher rates of suicide attempts among females, males are more likely to die by suicide. On the other hand, although we recruited patients from diverse geographic Spanish regions, the generalizability of these findings may be limited to hospitals that serve patients with similar sociodemographic and clinical characteristics. Finally, enrollment commenced in early 2021, during a period of COVID-19 public health restrictions and intermittent lockdowns. In addition to periodic recruitment closures, we cannot know the impact of the COVID-19 pandemic on participant flow and eligibility. Despite these limitations, this study has several notable strengths. It is the first in Spain to test a brief psychological intervention targeting adolescents with recent suicide attempts and is a significant advancement in the management of this high-risk population. In addition to being an RCT, it recruited a large sample of high suicide risk adolescents from diverse Spanish regions. Moreover, the study employed a proactive approach to minimize loss to follow-up. Despite some participant attrition, we employed a proactive approach and supplemented missing data with information from electronic health records. To ensure the quality, consistency, and proper implementation of the study, we provided participating therapists with structured training, and they participated in monthly meetings. Finally, in addition to evaluating the effectiveness of the intervention, the study also identified critical predictors of suicide reattempts, such as suicidal intentionality and impulsivity, providing valuable insights to guide future interventions.

## Conclusion

Preventing suicide in adolescents is a critical public health concern worldwide. Empirical evidence supports the efficacy of school-based public preventive interventions (i.e., YAM program), in reducing suicidal behaviors among adolescents in community samples [55]. However, this does not necessarily indicate that YAM-inspired brief interventions targeting clinical samples of adolescents at high suicide risk yield similar results.

Our multicenter RCT represents a pioneering effort to develop a brief and specific intervention for adolescents at high risk of suicide reattempts. Despite SAM not demonstrating superior efficacy compared to TAU, the findings provide initial evidence for SAM’s potential to reduce suicide reattempts in this vulnerable population. Notably, our results suggested that certain dimensions of impulsivity, such as attentional and nonplanning, may modulate the intervention’s effectiveness. While these findings were not part of the original hypotheses and require further confirmation, they may help guide the refinement of SAM toward more personalized approaches that address specific cognitive and behavioral profiles.

This study represents an important step forward in the search for innovative suicide prevention strategies. SAM could offer a practical and accessible psychological intervention alternative for mental health professionals, and hospitals could effectively integrate it into postdischarge care protocols. Personalized approaches that address distinct clinical profiles, such as impulsivity levels, could enhance the effectiveness of brief psychological interventions.

## Supporting information

10.1192/j.eurpsy.2025.10065.sm001 García-Fernández et al. supplementary material 1 García-Fernández et al. supplementary material

10.1192/j.eurpsy.2025.10065.sm002 García-Fernández et al. supplementary material 2 García-Fernández et al. supplementary material

## Data Availability

Access to de-identified individual participant data (including the data dictionary), statistical code, and other relevant materials is available upon reasonable request.

## Supplementary material

The supplementary material for this article can be found at http://doi.org/10.1192/j.eurpsy.2025.10065.
