# Supplementary material for: Efficacy of a brief psychological intervention for adolescents with recent suicide attempt: A randomized clinical trial
Source: Eur Psychiatry. 2025 Jul 10;68(1):e110. doi: 10.1192/j.eurpsy.2025.10065 (PMC12438991; doi:10.1192/j.eurpsy.2025.10065)
Supplement: García-Fernández et al. supplementary material 2 — García-Fernández et al. supplementary material [file S0924933825100655sup002.docx]

**Supplementary Table 2.** Quality Assessment of Controlled Intervention Studies

Study Quality Assessment Tools

| **Criteria** | **Yes** | **No** | **Other (CD, NR, NA)*** |
| --- | --- | --- | --- |
| 1. Was the study described as randomized, a randomized trial, a randomized clinical trial, or an RCT? | X |  |  |
| 2. Was the method of randomization adequate (i.e., use of randomly generated assignment)? | X |  |  |
| 3. Was the treatment allocation concealed (so that assignments could not be predicted)? | X |  |  |
| 4. Were study participants and providers blinded to treatment group assignment? |  | X |  |
| 5. Were the people assessing the outcomes blinded to the participants' group assignments? |  | X |  |
| 6. Were the groups similar at baseline on important characteristics that could affect outcomes (e.g., demographics, risk factors, co-morbid conditions)? | X |  |  |
| 7. Was the overall drop-out rate from the study at endpoint 20% or lower of the number allocated to treatment? | X |  |  |
| 8. Was the differential drop-out rate (between treatment groups) at endpoint 15 percentage points or lower? | X |  |  |
| 9. Was there high adherence to the intervention protocols for each treatment group? | X |  |  |
| 10. Were other interventions avoided or similar in the groups (e.g., similar background treatments)? | X |  |  |
| 11. Were outcomes assessed using valid and reliable measures, implemented consistently across all study participants? | X |  |  |
| 12. Did the authors report that the sample size was sufficiently large to be able to detect a difference in the main outcome between groups with at least 80% power? | X |  |  |
| 13. Were outcomes reported or subgroups analyzed prespecified (i.e., identified before analyses were conducted)? | X |  |  |
| 14. Were all randomized participants analyzed in the group to which they were originally assigned, i.e., did they use an intention-to-treat analysis? | X |  |  |

| **Quality Rating (Good, Fair, or Poor)** |
| --- |
| Self-reported rating. |
| Additional Comments (If POOR, please state why): |

*CD, cannot determine; NA, not applicable; NR, not reported
